# Differential Impact of Relative Dose-Intensity Reductions in Diffuse Large B-Cell Lymphoma Treated with R-CHOP21 or R-CHOP14

**DOI:** 10.1371/journal.pone.0123978

**Published:** 2015-04-24

**Authors:** Antonio Gutiérrez, Leyre Bento, Antonia Maria Bautista-Gili, Francesc Garcia, Jordi Martinez-Serra, Blanca Sanchez, Clara Martorell, Jordi Gines, Lucia Garcia, Eva Gimeno, Mariana Ferraro, Raquel Del Campo, Joan Bargay, Albert Perez, Javier Vercher, Miguel Scaff, Ana Pacheco, Carmen Ballester, Florencia Garcia, Rafael Ramos, Antonio Salar, Joan Besalduch

**Affiliations:** 1 Hematology Department, Son Espases University Hospital, Palma, Spain; 2 Hematology Department, Hospital del Mar, Barcelona, Spain; 3 Pharmacy Department, Son Espases University Hospital, Palma, Spain; 4 Hematology Department, Son Llatzer Hospital, Palma, Spain; 5 Hematology Department, Can Misses Hospital, Ibiza, Spain; 6 Hematology Department, Hospital of Manacor, Manacor, Spain; 7 Hematology Department, Mateu Orfila Hospital, Mahon, Spain; 8 Oncology Department, University Hospital Son Espases, Palma, Spain; 9 Pathology Department, University Hospital Son Espases, Palma, Spain; West German Cancer Center, GERMANY

## Abstract

DLBCL is an aggressive lymphoma treated with R-CHOP. Recently, attempts have been made to improve the outcome by increasing both dose-density and intensity but there have been no benefits in terms of survival. When treating malignancies RDI is important to consider but there is little published information on DLBCL. The purpose of this study was to analyze the differential prognostic impact of RDI in two cohorts of DLBCL patients treated with R-CHOP21 or R-CHOP14. From January 2001 to August 2013 we included DLBCL patients homogenously treated with R-CHOP21 or R-CHOP14, with or without radiotherapy, at University Hospital Son Espases, Hospital Son Llatzer of Palma and Hospital del Mar of Barcelona (N = 157). In order to avoid selection bias the patients were retrospectively identified from the Pathology Department and Pharmacy registries. Median follow-up was 68 months. There was no difference in the response or survival between the two cohorts. In the R-CHOP21 group, both a reduction higher than 15% in RDI (RR 7.41) and R-IPI (RR 2.99) were independently associated with OS. However, a reduction higher than 15% in RDI (RR 4.41) was only noted for PFS. In the R-CHOP14 group, NCCN-IPI (RR 7.09) and B-symptoms (RR 5.37) for OS; AA stage III-IV (RR 6.26) and bulky disease (RR 4.05) for PFS. There was a trend towards a higher rate of RDI reduction observed in the R-CHOP14 group but it only made an impact in the R-CHOP21 group. We conclude that R-CHOP21 and R-CHOP14 are equivalent regimens in terms of response and survival, but only if RDI reductions are avoided. For patients receiving R-CHOP21 we recommend using clinical and support measures in order to avoid RDI reductions.

## Introduction

DLBCL is the most common non Hodgkin lymphoma. It is an aggressive but potentially curable lymphoma [1]. Before the chemo-immunotherapy era, combination chemotherapy with cyclophosphamide, doxorubicin, vincristine and prednisone (CHOP) administered every 21 days was the established standard treatment.

More recently attempts have been made to improve the outcome by both increasing dose-density (DD) (CHOP14) or intensity (second and third generation regimens, CHOEP, ACVBP, frontline high dose therapy followed by autologous stem cell transplantation)[2, 3]. Only CHOP14 first and, more importantly, the addition of rituximab, has improved survival in comparison to standard CHOP [4–6].

Even though phase 2 studies had predicted promising results after adding rituximab to the regimen, when randomised phase 3 trials were carried out there were no shown benefits due to their higher toxicity when compared with R-CHOP[7, 8]. This has meant that R-CHOP administered every 21 days (R-CHOP21) has become the standard treatment for DLBCL patients.

Prognostic factors in DLBCL may be related to the patient (e.g. age and performance status), to the tumor itself and the aggressiveness of its markers (e.g. stage, tumor burden, proliferation index, LDH or beta-2-microglobulin) and to the therapeutic strategy (e.g. therapeutic regimen or relative dose intensity (RDI)). In routine clinical practice patient and tumor-related prognostic factors summarized in prognostic models such as the International Prognostic Index (IPI) and age-adjusted IPI (a-IPI) are considered[9]. A revised version was reported in the post-rituximab era[10] and a new enhanced version called NCCN-IPI, demonstrating a better discrimination for risk groups, has been recently reported[11].

However, treatment-related factors such as RDI are not always routinely considered. RDI represents the ratio of the amount of a drug actually administered to the amount planned for a fixed time period and is an important issue to consider when treating malignancies[12, 13]. The purpose of calculating RDI is to evaluate whether or not the planned dose intensity of a chemotherapy treatment was actually achieved. Although it is a well-known prognostic factor in Hodgkin lymphoma [14, 15], limited information has been published on DLBCL [16–18]. The purpose of this study is to further analyze the prognostic impact of RDI in two cohorts of DLBCL patients treated with R-CHOP21 or R-CHOP14 to evaluate its differential impact when increasing dose density.

## Methods

### Patients

All patients diagnosed with DLBCL from January 2001 to August 2013 at University Hospital Son Espases were retrospectively identified by the Pathology Department registry to avoid selection bias. Only patients treated with R-CHOP21 or R-CHOP14 +/- radiotherapy were included. We also added all the patients treated with R-CHOP14 during the same time period in two additional hospitals (Hospital Son Llatzer of Palma and Hospital del Mar of Barcelona) identified by their Pathology and Pharmacy registries to avoid selection bias.

Patients receiving other chemotherapy regimens or consolidations, with severe concomitant medical or psychiatric illnesses, central nervous system involvement or a bilirubin level >1.5 mg/dl, a cardiac ejection fraction of <50% and a pulmonary function test and diffusing lung capacity of <50% of the predictive value, were excluded. Double hit DLBCL were also excluded. The retrospective study was approved by the local ethics committee: Comitè ètic de d’Investigació clínica de les Illes Balears (CEIB-IB) with the number IB 1680/11 PI. Written inform consent were obtained from living patients. For those without written consent, patient records and information was anonymized and de-identified prior to analysis.

At diagnosis the main prognostic factors in DLBCL were obtained, including international prognostic index (IPI) factors[9] and NCCN-IPI[11]. Evaluations were carried out following standard guidelines[19].

### RDI calculation and statistical analysis

RDI represents the ratio of the amount of a drug actually administered to the amount planned for a fixed time period. RDI was calculated as previously described. Briefly, the RDI of each drug was obtained followed by an average of RDI in CHOP consisting in the sum of RDI of the 3 drugs divided by 3[12, 20].

Overall survival (OS) and progression-free survival (PFS) were measured from the date of diagnosis and were estimated according to the Kaplan-Meier method[21]. Comparisons among those variables of interest were performed by the log-rank test[22]. Multivariate analysis with the variables that proved to be significant in univariate analysis was performed according to the Cox proportional hazard regression model[23]. All p-values reported were two-sided and statistical significance was defined at p <0.05.

## Results

### Patient selection and clinical characteristics

A total of 188 patients were diagnosed and treated at Son Espases University Hospital in the time period selected from Pathology Department records. Seventy three patients were excluded for the following reasons: not receiving rituximab (n = 29), receiving additional consolidation therapy such as autologous stem cell transplantation or rituximab maintenance (n = 25), other regimens with higher or lower intensity (n = 9), move to another center (n = 6), diagnosis at autopsy (n = 2) and other reasons (n = 2). We included 115 patients (74 in the R-CHOP21 and 41 in the R-CHOP14 cohort) from Son Espases Hospital. We made the same process and selection in both Son Llatzer Hospital and Hospital del Mar, and included 24 and 18 patients, respectively, for a final total of 83 patients in the R-CHOP14 cohort. Table 1 shows that both cohorts were similar in the majority of clinical characteristics (n = 157), with the R-CHOP14 cohort being younger.

**Table 1:** Main clinical characteristics of the patients.

	R-CHOP21	R-CHOP14	P
	group (n = 74)	group (n = 83)	
**Age (median & range)**	65 (25–88)	55 (15–79)	0.001
**Sex (M/F)**	34 (46%) / 40 (54%)	51 (61%) / 32 (39%)	0.056
**ECOG PS > 1**	17 (23%)	18 (22%)	0.85
**Ann Arbor stage III-IV**	40 (54%)	48 (58%)	0.75
**B-symptoms**	25 (34%)	26 (31%)	0.86
**Elevated LDH**	33 (46%)	40 (49%)	0.75
**> 1 extranodal site**	8 (11%)	19 (23%)	0.057
**Bulky disease**	23 (31%)	34 (41%)	0.24
**a-IPI > 1**	31 (42%)	31 (38%)	0.63
**R-IPI unfavorable**	24 (32%)	26 (32%)	1
**NCCN-IPI:**			0.31
**- Low**	9 (13%)	17 (21%)	
**- Low-intermediate**	27 (39%)	35 (44%)	
**- High-intermediate**	26 (38%)	24 (30%)	
**- High**	7 (10%)	4 (5%)	
**Elevated**	32 (49%)	31 (39%)	0.24
**Beta-2-microglobulin**			
**Radiotherapy**	27 (36%)	27 (32%)	0.62

R-CHOP: rituximab, cyclophosphamide, doxorubicin, vincristine, prednisone; M: male, F: female; ECOG PS: Eastern Cooperative Oncology Group performance status; LDH: lactate dehydrogenase; a-IPI: age-adjusted International Prognosis Index. R-IPI: revised International Prognosis Index; NCCN-IPI: enhanced international prognostic index.

### Treatment and dose intensity reductions

Median number of cycles of R-CHOP received was 6 in both groups (range 1–8). Patients in the R-CHOP14 cohort received primary G-CSF prophylaxis, whereas in the R-CHOP21 41% of patients received secondary G-CSF prophylaxis. We reserved primary prophylaxis for the 28% of the patients with more than a 20% risk of febrile neutropenia following standard guidelines for G-CSF use [24, 25]. Thirty percent of the patients in the R-CHOP21 group needed no G-CSF support. In both groups, 36% and 32% of patients were consolidated with radiotherapy, respectively for R-CHOP21 and R-CHOP14 (p = 0.62).

In spite of the above presented differential G-CSF prophylaxis policy, median reduction in RDI showed a tendency to be higher in the R-CHOP14 cohort: 9.2% versus 5.8% for R-CHOP21 (p = 0.094). However, the percentage of patients with a reduction in RDI greater than 15% between the two cohorts was similar (34% and 31% for R-CHOP14 and R-CHOP21 groups, respectively; p = 0.74). The causes for a higher than 15% reduction in the R-CHOP14 cohort (n = 28) were: infectious complications in 14 patients (48%), hematological toxicity in 6 (21%), age and comorbidities in 2 (7%), neurotoxicity in 2 (7%), poor compliance or logistic problems in 2 (7%) and other non-related causes in 2 (7%). The causes for a higher than 15% reduction in the R-CHOP21 cohort (n = 23) were: age and comorbidities in 12 (52%), hematological toxicity in 4 (17%), infectious complications in 2 (9%), mucositis in 2 (9%), poor compliance in 1 (4%) and other non-related causes in 2 (9%).

### Outcome and survival analysis between treatment groups

Overall response and complete response rates were similar in both groups: 86% and 76% for R-CHOP21 and 94% and 74% for R-CHOP14 (p = 0.17 and p = 0.85, respectively). However, when we considered only patients with higher than 15% reduction in RDI (n = 49), a significant reduction in CR rate was observed in the R-CHOP21 compared to the R-CHOP14 cohort (52% versus 82%, respectively; p = 0.033).

The median follow-up for living patients was 68 months (4–156). Tables 2–4 show the univariate and multivariate analysis of prognostic factors influencing OS and PFS.

**Table 2:** Prognostic factors influencing OS and PFS in the global group.

	**5y-OS**	**P**	**5y-PFS**	**P**
**Age:**		0.001		0.047
- 0–60	86% (78–94)		80% (72–89)	
- >60	65% (54–77)		67% (55–79)	
**Sex:**		0.58		0.9
- Male	78% (69–88)		75% (65–85)	
- Female	73% (62–84)		72% (61–84)	
**ECOG PS:**		0.001		0.1
- 0–1	80% (73–88)		77% (69–85)	
- 2–4	60% (44–76)		63% (45–81)	
**Ann Arbor stage:**		0.33		0.006
- I-II	79% (69–89)		86% (78–94)	
- III-IV	74% (64–84)		64% (53–65)	
**B-symptoms:**		0.008		0.008
- No	83% (75–90)		81% (73–89)	
- Yes	62% (48–76)		57% (42–73)	
**LDH:**		0.15		0.057
- Normal	84% (75–92))		80% (70–90)	
- Elevated	67% (56–78)		67% (56–79)	
**Extranodal sites:**		0.47		0.47
- 0–1	78% (65–92)		75% (67–83)	
- > 1	65% (46–83)		71% (53–89)	
**Bulky disease:**		0.87		0.18
- No	77% (68–85)		76% (67–85)	
- Yes	75% (63–86)		70% (58–83)	
**Beta-2-microglobulin:**		0.012		0.2
- Normal	85% (77–93)		80% (71–89)	
- Elevated	70% (58–82)		71% (59–84)	
**R-IPI:**		0.011		0.001
- Low-int	83% (76–91)		83% (75–90)	
- High	60% (46–74)		56%(40–71)	
**NCCN-IPI:**		<0.001		0.010
- Low-int	78% (71–85)		75% (67–82)	
- High	34% (5–63)		48% (11–85)	
**Radiotherapy:**		0.21		0.25
- Yes	82% (71–93)		80% (69–91)	
- No	73% (64–82)		70% (61–80)	
**RDI reduction:**		0.002		0.001
- 0–15%	83% (75–91)		82% (74–90)	
- >15%	62% (48–75)		57% (42–72)	
**Treatment group:**		0.45		0.51
- RCHOP14	80% (71–89)		76% (67–86)	
- RCHOP21	70% (59–82)		70% (58–82)	
**Multivariate analysis**				
	**Overall survival**		**Progression-free survival**	
**Parameter**	**RR (95% CI)**	**P**	**RR (95% CI)**	**P**
**Age > 60 years**	3.19 (1.56–6.53)	0.002	—-	—-
**RDI reduction >15%**	2.63 (1.32–5.26)	0.006	2.42 (1.24–4.69)	0.009
**ECOG PS > 1**	2.25 (1.12–4.5)	0.022	—-	—-
**Unfavorable R-IPI**	—-	—-	2.62 (1.35–5.09)	0.005

OS: overall survival; PFS: progression-free survival; ECOG PS: Eastern Cooperative Oncology Group performance status; LDH: lactate dehydrogenase; a-IPI: age-adjusted international prognosis index; R-IPI: revised International Prognosis Index; NCCN-IPI: enhanced International Prognostic Index; RDI: relative dose-intensity; R-CHOP: rituximab, cyclophosphamide, doxorubicin, vincristine, prednisone.

**Table 3:** Prognostic factors influencing OS and PFS.

	R-CHOP14 group				R-CHOP21 group			
	5y-OS	P	5y-PFS	P	5y-OS	P	5y-PFS	P
**Age:**		0.057		0.2		0.006		0.17
- 0–60	86% (76–96)		80% (69–91)		86% (70–100)		81% (61–100)	
- >60	71% (54–87)		70% (52–87)		62% (47–77)		64% (49–79)	
**Sex:**		0.92		0.86		0.63		0.85
- Male	80% (69–91)		78% (66–90)		74% (57–92)		71% (55–87)	
- Female	80% (66–95)		74% (59–90)		67% (52–83)		70% (54–87)	
**ECOG PS:**		0.021		0.19		0.032	61% (47–74)	0.34
- 0–1	84% (74–93)		79% (69–89)		75% (62–89)		73% (55–90)	
- 2–4	67% (45–88)		66% (42–90)		53% (29–77)		61% (47–74)	
**Ann Arbor stage:**		0.09		0.01		0.82		0.17
- I-II	85% (73–97)		91% (81–100)		72% (56–87)		81% (54–100)	
- III-IV	76% (64–88)		65% (51–80)		68% (50–87)		60% (46–74)	
**B-symptoms:**		<0.001		0.005		0.88		0.39
- No	91% (83–98)		85% (75–95)		72% (59–86)		76% (57–95)	
- Yes	57% (38–76)		55% (34–76)		65% (43–88)		58% (34–81)	
**LDH:**		0.24		0.057		0.41		0.48
- Normal	90% (80–99)		84% (72–96)		76% (61–91)		75% (59–91)	
- Elevated	69% (55–84)		70% (54–85)		62% (44–81)		64% (45–82)	
**Extranodal sites:**		0.14		0.27		0.73		0.93
- 0–1	85% (77–94)		78% (67–88)		70% (58–83)		70% (52–89)	
- > 1	63% (41–85)		71% (49–92)		71% (38–100)		73% (41–100)	
**Bulky disease:**		0.45		0.049		0.65		0.95
- No	85% (75–95)		82% (71–94)		67% (52–82)		68% (53–83)	
- Yes	73% (58–88)		67% (50–84)		78% (61–95)		75% (56–94)	
**Beta-2-microglobulin:**		0.38		0.46		0.008		0.27
- Normal	83% (72–94)		78% (66–90)		89% (78–100)		83% (70–97)	
- Elevated	77% (62–92)		74% (58–91)		61% (41–80)		65% (45–86)	
**R-IPI:**		0.18		0.010		0.023		0.022
- Low-int	87% (78–96)		85%(75–95)		78% (65–91)		81% (69–92)	
- High	65% (47–84)		60% (40–80)		55% (33–76)		49% (25–73)	
**NCCN-IPI:**		<0.001		0.014		0.059		0.18
- Low-int	82% (74–91)		78% (68–87)		72% (59–85)		70% (57–83)	
- High	25% (0–67)		50% (0–100)		38% (0–77)		44% (0–88)	
**Radiotherapy:**		0.16		0.79		0.73		0.18
- Yes	85% (72–98)		78% (62–93)		77% (60–95)		82% (65–98)	
- No	78% (66–89)		75% (63–87)		66% (51–82)		64% (48–79)	
**RDI reduction:**		0.82		0.12		<0.001		<0.001
- 0–15%	79% (68–90)		82% (71–93)		88% (77–98)		82% (71–94)	
- >15%	82% (67–96)		65% (47–84)		37% (27–48)		43% (19–67)	

R-CHOP: rituximab, cyclophosphamide, adriamycin, vincristine, prednisone. OS: overall survival. PFS: progression free survival. ECOG PS: Eastern Cooperative Oncology Group Performance Status. LDH: lactate dehydrogenase. R-IPI: revised international prognosis index. NCCN-IPI: an enhanced international prognostic index. RDI: relative dose-intensity.

**Table 4:** Multivariate analysis.

**R-CHOP21 cohort**						
	**Overall survival**			**Progression-free survival**		
**Parameter**	**p**	**RR (exp. B)**	**95% CI**	**p**	**RR (exp. B)**	**95% CI**
**RDI reduction >15%**	<0.001	7.41	2.51–21.83	0.001	4.41	1.77–10.99
**Unfavourable R-IPI**	0.032	2.99	1.1–8.16	—	—	—
**R-CHOP14 cohort**						
	**Overall survival**			**Progression-free survival**		
**Parameter**	**p**	**RR (exp. B)**	**95% CI**	**p**	**RR (exp. B)**	**95% CI**
**High NCCN-IPI**	0.005	7.09	1.83–27.42	—	—	—
**B-symptoms**	<0.001	5.37	2.11–13.7	—	—	—
**Ann Arbor stage**	—	—	—	0.004	6.26	1.77–22.13
**Bulky disease**	—	—	—	0.005	4.05	1.54–10.67

R-CHOP: rituximab, cyclophosphamide, adriamycin, vincristine, prednisone. RDI: relative dose-intensity. RR: relative risk. R-IPI: revised international prognosis index. NCCN-IPI: an enhanced international prognostic index.

**Fig 1:**
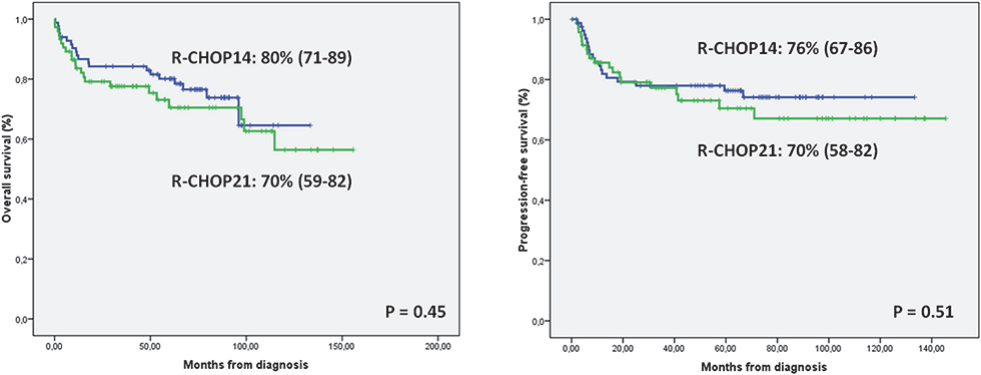
5-year OS and PFS in R-CHOP21 and R-CHOP14 cohorts.

When considering the global group (Table 2), there were no differences between the two cohorts in terms of either OS or PFS (Fig 1). A reduction higher than 15% in RDI was the only factor independently associated with both a worse OS (RR 2.63) and PFS (RR 2.42). Older age (RR 3.19) and ECOG PS>1 (RR 2.25) also independently influenced OS, and an unfavorable R-IPI was also associated with a worse PFS (RR 2.62).

**Fig 2:**
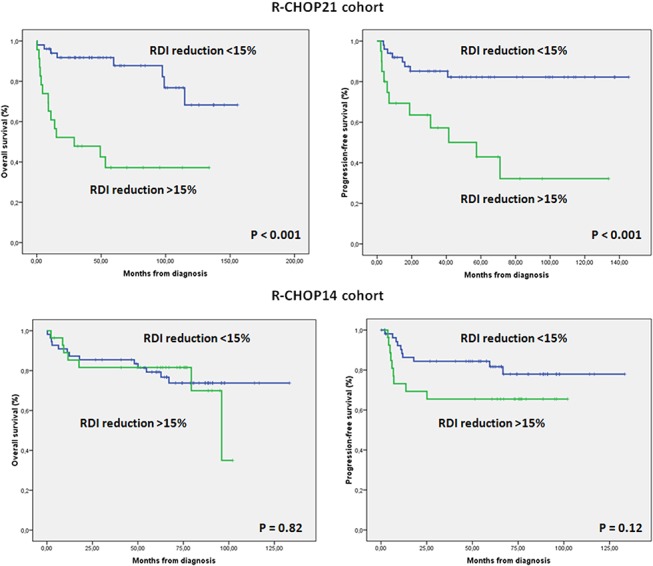
Diferential impact of RDI reduction in R-CHOP21 and R-CHOP14 cohorts.

In R-CHOP21 (Table 4), both a reduction higher than 15% in RDI (RR 7.41) and an unfavorable R-IPI (RR 2.99) were independently associated with a worse OS. For PFS only a reduction higher than 15% in RDI (RR 4.41) was independently associated with worse PFS (Fig 2). By contrast, in the R-CHOP14 group an unfavorable NCCN-IPI (RR 7.09) and the presence of B-symptoms (RR 5.37) was independently associated with worse OS, and a AA stage III-IV (RR 6.26) and bulky disease (RR 4.05) were independently related to worse PFS. RDI reductions showed no statistical significance on OS or PFS in patients treated with R-CHOP14. However there was a mild trend towards a better PFS in patients with RDI reduction lower than 15%. This may suggest that in R-CHOP14 it is also an important factor but that patients treated with R-CHOP14 have a wider margin of security associated with its higher dose-density (Fig 2).

## Discussion

R-CHOP21 remains the standard of care for patients with DLBCL. In recent phase III trials an increase in dose-density did not lead to a better outcome. There were no differences observed in terms of response, PFS or OS between R-CHOP21 and R-CHOP14 [7, 8]. In our series we also found no differences, with both regimens showing a similar outcome (Fig 1).

Furthermore, R-CHOP14 proved to be more toxic and there were more difficulties in maintaining RDI in the GELA study. In our series, RDI reductions showed a tendency to be higher in the R-CHOP14 cohort (9.2% versus 5.8% in the R-CHOP21 cohort) (p = 0.094). Delarue et al. reported a median RDI reduction of around 12% for R-CHOP14, and 2% for R-CHOP21 (p<0.001)[8]. Another British trial reported similar median RDI between groups [7]. However, in both trials the R-CHOP14 median RDI of all important drugs relative to R-CHOP21 patients was 134% and 150%, respectively. With both regimens having similar effectiveness, the direct consequence is that RDI reductions seem to have much more relevance in R-CHOP21 than in R-CHOP14.

RDI is an important issue in oncology. Early studies analyzed its role in breast cancer and other malignancies such as Hodgkin lymphoma[26, 27]. Dose intensity (DI) represents the amount (mg/m2) of a drug administered per unit time (week) and RDI reflects whether DI was implemented as planned. Multiple works have reported a correlation between RDI and survival prognosis.

In 1990 the impact of RDI in DLBCL was described using CHOP regimen or others with higher intensity[16–18]. After the addition of rituximab to the CHOP scheme (R-CHOP), another report described that mortality was affected by RDI[20]. In these works several cutoffs were used to define RDI reduction: 25%, 15% and median RDI. In our analysis we selected 15%, as this is the most used in RDI studies, both in lymphoma and solid malignancies. Nevertheless, we also tried several cutoffs and finally this was the best one for balancing clinical value and statistical potency.

As expected, in our series RDI reductions only made an impact on the R-CHOP21 group but not R-CHOP14 (Fig 2). Moreover, in the R-CHOP21 group, a greater than 15% reduction in RDI was independently associated with both OS and PFS, with R-IPI also independently influencing OS while RDI reductions had no impact in the R-CHOP14 group. In fact, patients, with and without a greater than 15% reduction, had 82% and 79% 5y-OS (p = 0.82) and 65% and 82% 5y-PFS (p = 0.12), respectively.

Nonetheless, in most large-scale clinical trials RDI tends to remain satisfactory. However, for several reasons in routine clinical practice it is not always easy to maintain RDI. The first step to manage this problem is to realize that the problem exists, and the second is to analyze the causes and potential solutions. We demonstrate that in the past in our center we have had this problem, as the 31% of patients that suffered greater than 15% of RDI reduction fared worse than patients without this reduction in patients treated with R-CHOP21 but not for the 34% with the same reduction that received R-CHOP14 (in either our center or the other two participating hospitals). Furthermore, this was independent of standard clinical prognostic factors.

The reasons for these RDI reductions were: advanced age, comorbidities, hematologic toxicity or complications such as infections, patient’s social factors or calendar conflicts. In our R-CHOP21 cohort half of these reductions were related to age and comorbidities but also to toxic events including infections, which may be anticipated or carefully monitored. Older patients often do not receive the same dose intensity or density as young patients. Lyman et al. reported several independent prognostic factors of dose-intensity reduction: age older than 60 years, advanced disease stage, poor performance status, and no prophylactic G-CSF use[28]. Interestingly, age was no longer a significant risk factor in patients who received G-CSF.

With this in mind, and considering the risk of hematologic toxicity, predictive models based on the identified risk factors for reduced RDI should be considered to decide the appropriate supportive care, including administration of hematopoietic growth factors, which facilitates the delivery of full chemotherapy dose. Zelenetz [29] identified older age, low albumin, presence of hepatic dysfunction, bone marrow involvement by the disease and/or low neutrophil at diagnosis as predicting factors for high risk of neutropenia in the first cycle of chemotherapy and thereby potential risk for lower dose intensity. Intragumtornchai et al. [30] have proposed another model for predicting life-threatening neutropenia after the first course of CHOP in aggressive NHL without taking age into account. In this model, the presence of low albumin levels, high LDH and bone marrow involvement by the lymphoma predicted a 72% rate of febrile neutropenia with full dose of CHOP as the therapeutic regimen.

Other studies focused on the possibility of delivering full doses of active drugs in older patients by using growth factors assuming that this may have an impact on the outcome [4, 31]. By taking into consideration all this evidence, guidelines have been developed that recommend the use of G-CSF in older patients receiving treatment with intention to cure [24, 25]. Growth factors should be administered to patients receiving chemotherapy associated with febrile neutropenia (≥20%) or to patients for whom reductions in chemotherapy dose density or intensity are known to be associated with poor prognosis.

Doxorubicin is a key drug in the treatment of this type of lymphoma but it is often withheld from older patients because of its cardiotoxicity [32]. Hershman et al. [33] reported that older patients with DLCL receiving doxorubicin were more likely to develop chronic heart failure, with hypertension being the only synergistic risk factor with this drug. Less cardiotoxic formulations of doxorubicin, such as liposomal doxorubicin, may be less cardiotoxic and with a similar outcome [34].

Patient’s social factors or calendar conflicts may generate RDI reductions. Some associated with the patient and others to the Health System: waiting lists and work overload exceeding system capacity. In both cases the key is that patients and physicians recognize the importance of RDI in DLBCL treated with R-CHOP21.

Overall in our series there were no differences in terms of response or survival between patients treated with R-CHOP21 or R-CHOP14. A tendency towards a higher rate of RDI reduction was observed in the R-CHOP14 group. However, the impact of RDI reductions on response and survival was only observed in the R-CHOP21 group but not in patients treated with R-CHOP14. We conclude that, if RDI reductions are avoided, R-CHOP21 and R-CHOP14 are equivalent regimens in terms of response and survival. For patients receiving R-CHOP21 we recommend using clinical and support measures in order to avoid RDI reductions.
